# 

**Published:** 1850-08

**Authors:** 


### WESTERN JOURNAL

OF

### MEDICINE AND SURGERY.

EDITORS.

---

**LUNSFORD P. YANDELL, M. D.**

PROFESSOR OF PHYSIOLOGY AND PATHOLOGICAL ANATOMY IN THE UNIVERSITY OF LOUISVILLE.

AND

**THEODORE S. BELL, M.D.**

FOREIGN CORRESPONDENCE.

Our readers will recognise in the initials of our foreign correspondent, a signature that has pleased, instructed, and charmed them in a former number of this Journal. The writer will continue his labors.

The WESTERN JOURNAL OF MEDICINE and SURGERY is published monthly by the undersigued, at the corner of Main and Fifth streets, Louisville, at $5 per annum, payable in advance. Each number contains 96 pages, making two volumes in the year of more than 550 pages each.

Contributions to its pages by the Physicians of the Valley of the Mississippi addressed tothe Editors, are respectfully solicited.

Letters on business, to be addressed, postage paid, to the publishers.

Jauuary, 1844. PRENTICE & CO.

### *Receipts of Medical Journal for July*, 1850.

| | |
|---|---|
| Dr. W. White, Glasgow, Ky., | $10 00 |
| R. P. Tully, Helena, Ky., | 10 00 |
| D. E. Gibson, Bellmont Tenn., | 5 00 |
| A. G. Stevenson. Kirksville, Ohio, | 9 00 |
| G. D. Morrow, Savannah, Tann., | 10 00 |
| G. W. Payne, Preston, Miss., | 5 00 |
| W. J. Holmes, McAllister's Cross Roads, Tenn., | 5 00 |
| J. Davidson, Petersburg, Ind., | 5 00 |
| G. W. Gamble, Pinckneyville, Ala., | 10 00 |
| T. C. Osborne, Frie, Ala., | 5 00 |
| M. H. Jackson, Versailles, Tenn., | 5 00 |
| J. M. Sharp, [illegible]onconnah, Tenn., | 10 00 |
| D. Pierce, Dresden, Ohio, | 15 00 |
| R. P. Hunt, Lexington, Ky., | 2 50 |

